# Preparation and Thermoelectric Properties Study of Bipyridine-Containing Polyfluorene Derivative/SWCNT Composites

**DOI:** 10.3390/polym11020278

**Published:** 2019-02-07

**Authors:** Chengjun Pan, Luhai Wang, Wenqiao Zhou, Lirong Cai, Dexun Xie, Zhongming Chen, Lei Wang

**Affiliations:** 1 Shenzhen Key Laboratory of Polymer Science and Technology, College of Materials Science and Engineering, Shenzhen University, Shenzhen 518060, China; wangluhai0625@163.com (L.W.); zhou-wq@foxmail.com (W.Z.); 2 School of Environment and Civil Engineering, Dongguan University of Technology, Dongguan 523808, China; clr@dgut.edu.cn; 3 School of Chemistry, Sun Yat-sen University, Guangzhou 510275, China; xiedx9@mail.sysu.edu.cn; 4 Shenzhen Research Institute, Sun Yat-sen University, Shenzhen 518057, China

**Keywords:** organic thermoelectric, composites, SWCNTs, bipyridine, transition metal complex

## Abstract

Polymer/inorganic thermoelectric composites have witnessed rapid progress in recent years, but most of the studies have focused on the traditional conducting polymers. The limited structures of traditional conducting polymers restrain the development of organic thermoelectric composites. Herein, we report the preparation and thermoelectric properties of a series of composites films based on SWCNTs and bipyridine-containing polyfluorene derivatives. The value of the power factor around 12 μW m^−1^ K^−2^ was achieved for the composite F8bpy/SWCNTs with a mass ratio of 50/50, and the maximum value of 62.3 μW m^−1^ K^−2^ was obtained when the mass ratio reached 10/90. Moreover, taking advantage of the bipyridine unit could chelate various kinds of metal ions to form polymer complexes. The enhanced power factor of 87.3 μW m^−1^ K^−2^ was obtained for composite F8bpy-Ni/SWCNTs with a mass ratio of 50/50. Finally, the thermoelectric properties of the bipyridine-containing polyfluorene derivative/SWCNT composites were conveniently tuned by chelating with different metal ions.

## 1. Introduction

The exploitation of sustainable and environmentally friendly energy materials has been a hot topic due to the gradual exhaustion of fossil energy [1]. Thermoelectric materials have the ability to directly convert between heat and electricity by the motion of an internal carrier. As a result, wasted heat from daily activity and industrial productions were converted to electricity [2,3,4]. The dimensionless figure of merit *ZT* is usually used to evaluate the conversion efficiency of thermoelectric materials, *ZT* = *S*^2^*σT/κ*, where *σ*, *S*, *T*, and *κ* are the electrical conductivity, Seebeck coefficient, absolute temperature, and thermal conductivity, respectively [5,6,7]. Another parameter is mainly used for organic thermoelectric materials due to their very low thermal conductivity (usually between 0.1 and 0.5 W m^−1^ K^−1^) [8,9]. In the past several years, great progress has been made in the development of inorganic thermoelectric materials, such as skutterudites, bismuth telluride, and Zintl phases, which exhibited good thermoelectric performance [10,11]. Compared to inorganic thermoelectric materials, organic thermoelectric materials have a number of distinctive advantages including their lightweight material, flexibility, processability, low cost, and regulated molecular structure, which recently received significant attention in the field of thermoelectric materials [12,13,14].

Some intrinsically conductive polymers (ICPs) show excellent thermoelectric properties after doping. Pipe et al. reported a high *ZT* value of 0.42 for poly(3,4-ethylenedioxythiophene) (PEDOT) after it was doped with poly(styrenesulfonate) (PSS) [15]. Shi et al. explored an n-type conjugated polymer FBDPPV with the highest power factor of 28 μW m^−1^ K^−2^ in a solution with processable n-type conjugated polymers [16]. Fan et al. enhanced the thermoelectric properties of PEDOT:PSS greatly by ion accumulation of an ionic liquid on the surface of a polymer [17]. Otherwise, some conjugated polymers used in the polymer solar cells field usually have high mobility. High mobility benefits the improvement of thermoelectric properties of conjugated polymers. Some conjugated polymers used in the polymer solar cells field have been used to explore applications in organic thermoelectric materials, and some good results have been achieved [18,19,20,21,22]. However, due to their low electrical conductivity, the thermoelectric properties of ICPs are still not ideal.

Conductive polymer composites (CPCs), taking advantage of the good electric conductivity of inorganic materials and the large Seebeck coefficient of organic materials, exhibited enhanced thermoelectric performance compared to each single component [23,24,25,26]. Luo et al. realized the switching from p-type to n-type of polypropylene-based melt composites with SWCNTs by the addition of polyethylene glycol [27]. Cho et al. obtained high-performance CPC thin films consisting of polyaniline (PANi), graphene, double-walled nanotubes (DWNTs), and PEDOT:PSS by the layer-by-layer (LBL) method [28]. Rapid progress has been made in CPCs; however, the recently investigated conjugated polymer components are quite limited compared to the conventional conducting polymers, such as PEDOT, PANi, polypyrrole (PPy), polythiophene (PTh), and their derivatives [29,30,31,32,33,34]. It is an urgent task to develop new conjugated polymer systems with good thermoelectric performance to better understand the structure–thermoelectric performance relationship, thus furthering the development of polymer-based thermoelectric materials with good performance.

Bipyridine units containing organic molecules and conjugated polymers have been extensively applied to complex various transition metal ions, and play an important role in tuning the fluorescence properties of molecules [35]. Tuning the thermoelectric properties of composites by the complexion of transition metal ions into a polymer backbone has been reported by Chen et al. [36], but the influencing mechanism is still unclear, and thermoelectric performance still needs further improvement. In this paper, we report the preparation and thermoelectric properties of a series of composite films from a copolymer containing 9,9′-dioctyl-fluorene and bipyridine units and SWCNTs. We systematically investigated the effects of the different ratios of single-walled carbon nanotube loading and different types of transition metal ions on the thermoelectric properties of the composites.

## 2. Experimental Procedures

### 2.1. Raw Materials

The monomer 2,2′-(9,9-dioctyl-9H-fluorene-2,7-diyl) bis(4,4,5,5-tetra-methyl-1,3,2-dioxaborolane) was purchased from the Energy Chemical, Shanghai, China. 5,5′-dibromo-2,2′-bipyridine was purchased from Alfa Chemical Co., Ltd., Zhengzhou, China. SWCNTs (length: 5–30 μm, diameter: <3 nm, purity: >95.0 wt.%) were provided by XFNANO Materials Tech Co., Ltd. Nanjing, China. Tetrakis (triphenylphosphine) palladium (Pd(PPh_3_)_4_), potassium carbonate (K_2_CO_3_), manganese chloride (MnCl_2_), ferric chloride (FeCl_3_), cobalt chloride (CoCl_2_), nickel chloride (NiCl_2_), copper chloride (CuCl_2_), and zinc chloride (ZnCl_2_) were purchased from Energy Chemical, Shanghai, China. Toluene was dried with CaH_2_ prior to use. All other reagents, including distilled water, chlorobenzene (AR), methanol (AR), acetone (AR), hexane (AR), and chloroform (AR) were received and used without any further purification.

### 2.2. Preparation of F8bpy

**Figure 1:**
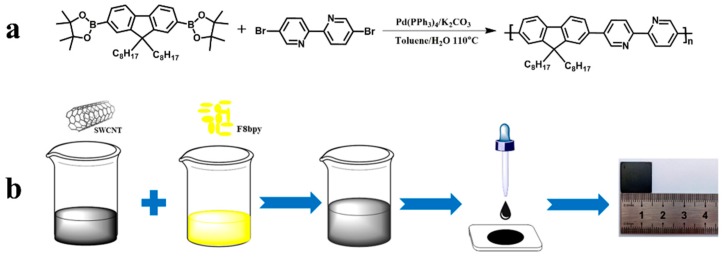
Synthesis of F8bpy (**a**), and schematic illustration showing the preparation process of the F8bpy or F8bpy/metal complex/SWCNT nanocomposites (**b**).

The polymer F8bpy (Figure 1a) was synthesized by Suzuki polymerization, a mixture of 2,2′-(9,9-dioctyl-9H-fluorene-2,7-diyl) bis (4,4,5,5-tetra-methyl-1,3,2-dioxaborolane) (867.0 mg, 1 mmol), 5,5′-dibromo-2,2′-bipyridine (314.0 mg, 1 mmol), Pd(PPh_3_)_4_ (34.7 mg, 0.03 mmol), and K_2_CO_3_ (1.38 g, 10 mmol) was dissolved in degassed toluene (8 mL) and H_2_O (2 mL) under a nitrogen atmosphere, after which the reaction was heated to reflux and kept for 72 h. After the reaction was completed and cooled to room temperature, the polymer was extracted with chloroform and H_2_O, the chloroform solvent was removed under reduced pressure, and the polymer was precipitated in methanol. Then the crude polymer was washed with methanol, acetone, and hexane to remove oligomers and impurities. Finally, the resulting polymer was dried at 40 °C overnight under a vacuum. The polymer was obtained as yellow powder *M*_n_: 25.5 K, PDI: 2.54, ^1^H NMR (600 MHz, CDCl_3_, *δ*): 9.04 (s, 2H), 8.58 (s, 2H), 8.13 (s, 2H), 7.87 (d, *J* = 6.0 Hz, 2H), 7.66 (d, *J* = 18 Hz, 6H), 2.09 (s, 2H), 1.62 (s, 2H), 1.42–0.99 (m, 16H), 0.90–0.62 (m, 14H).

### 2.3. Preparation of F8bpy/SWCNT Composite Films

The F8bpy/SWCNT composite films with different weight ratios of SWCNTs were prepared by mechanically mixing F8bpy and SWCNTs in chlorobenzene. The general preparation procedure is as follows. First, 20 mg of SWCNTs were dispersed in chlorobenzene (20 mL) and the mixture was stirred for 6 h to be well dispersed. Then, F8bpy (20 mg) was dissolved in chlorobenzene (5 mL). Finally, F8bpy in chlorobenzene was added to the dispersion of SWCNTs, and stirred for 12 h at room temperature. Composite films with 50 wt.% of SWCNTs were prepared by drop-casting the mixture on clean glass substrates (15 mm × 15 mm) under ambient conditions. After the solvent was evaporated naturally, composite films that could be used for testing were obtained. Other composite films with different contents of SWCNTs were prepared by a similar procedure.

### 2.4. Preparation of F8bpy/Metal Complex/SWCNT Composite Films

A solution of 1 M transitions metal salts such as MnCl_2_, FeCl_3_, CoCl_2_, NiCl_2_, CuCl_2_, and ZnCl_2_in methanol was prepared. Meanwhile, a solution of F8bpy (10 mM) in chlorobenzene was also prepared and separated into several small vials. The solution of the transition metal salt was added to the solution of F8bpy (with 50/50 molar ratios) via a syringe. The mixture was stirred for 6 h at room temperature. Then a dispersion of SWCNTs in chlorobenzene with a specific weight ratio was added to the F8bpy/transition metal complex solution. After stirring the mixture for 6 h, the mixture was drop-casted onto the clean glass substrate (15 mm × 15 mm) in order to form F8bpy/transition metal/SWCNT composite films after the solvent had evaporated naturally at room temperature.

### 2.5. Characterization of F8bpy and F8byp/Metal Complex and Their Composites Films

The chemical structure of F8bpy was verified by ^1^H NMR spectra, which were recorded using a Bruker ADVANCE III 600 MHz NMR spectrometer (Fällanden, Switzerland). Molecular weights and distributions of polymers were determined by using gel permeation chromatography (GPC) (Waters e2695 Separations Module), where polystyrene was used as the standard and THF was used as the eluent. An ultraviolet-visible (UV-vis) spectrum (Thermo Fisher Scientific, Waltham, MA, USA) was obtained by a Thermo Evolution 220 UV-vis spectrophotometer. The morphologies and energy dispersive X-ray spectroscopy (EDS) of the composite films were observed by using a field-emission scanning electron microscope (Hitachi SU-70, Tokyo, Japan). The electrical conductivities and the Seebeck coefficients of the composite film samples were measured by using a commercial device MRS-3 thin film thermoelectric test system (Wuhan Joule Yacht Science and Technology Co., Ltd., Wuhan, China). The photograph and testing principle of this device are shown in Figure S5.

## 3. Results and Discussion

### 3.1. Synthesis of F8bpy and the Preparation of F8bpy and F8byp/Metal Complex/SWCNT Composite Films

F8bpy was synthesized by using Suzuki polymerization. During the synthesis of the polymer, we found that the quality of Pd(PPh_3_)_4_ was the key factor to improve the molecular weight of polymers. The polymerization with fresh palladium catalyst was highly efficient and provided us with very high molecular weights (up to 25.5 K) (Figure S2). The chemical structure of the polymer was confirmed by ^1^HNMR (Figure S1). The obtained polymers are soluble in common organic solvents such as THF, DCM, chloroform, chlorobenzene, etc. The preparation procedure of the F8bpy or F8bpy/metal complex nanocomposites is depicted in Figure 1b. After mixing the polymers with SWCNTs, ultra-sonication was necessary to facilitate the interface interactions (such as the π–π interaction) between the polymers and SWCNTs. The homogeneity and thickness of the composite films could be tuned by the amount of solutions drop-casted onto the glass substrates. The thickness information of composite and SWCNT films is shown in Table S1; those composite films were found to be flexible. A photograph of the composite film (F8bpy/SWCNTs with a mass ratio of 50/50) in its bending state is shown in Figure S4.

### 3.2. Morphology of the F8bpy/SWCNT Composite Films

**Figure 2:**
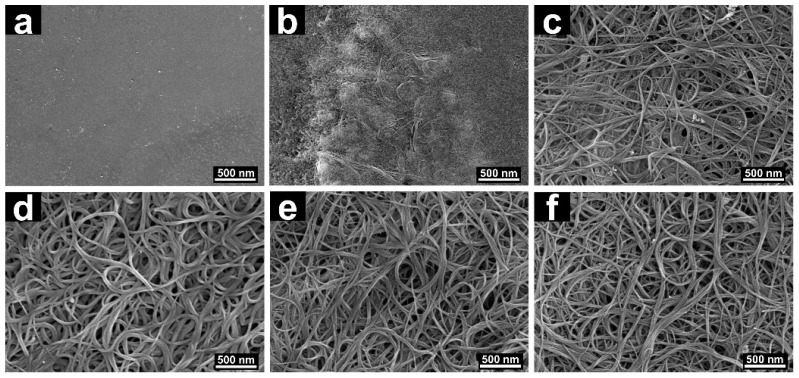
SEM images of F8bpy/SWCNT hybrid films with different SWCNT contents: 0 (**a**), 10 (**b**), 30 (**c**), 50 (**d**), 70 (**e**), and 90 wt.% (**f**).

The morphologies of F8bpy/SWCNT composites containing different mass ratios of SWCNTs were observed through FE-SEM, as images shown in Figure 2. As shown in Figure 2a, the relatively flat morphology was formed from the pure polymer F8bpy due to the weak interactions between polymer chains, which caused entanglement tightness of polymer chains. The high molecular weight of the polymer also promotes surface smoothing. The morphology of the composite with 10 wt.% SWCNTs is seen in Figure 2b. It can be observed that SWCNTs were wrapped in the polymer matrix, and a small amount of SWCNTs penetrated the surface of the polymer films. The homogeneous SWCNTs network could be observed from the SEM images of nanocomposites by increasing the amount of SWCNTs. The morphologies of one-dimensional wires is obvious, as shown in Figure 2c–f, and the diameter of the SWCNTs became larger due to the content of the increased bundled SWCNTs and the efficient encapsulation of F8bpy to the SWCNTs surface through π–π stacking or van der Waals forces between SWCNTs and F8bpy.

### 3.3. Characterizations of the F8bpy/Metal Complex

**Figure 3:**
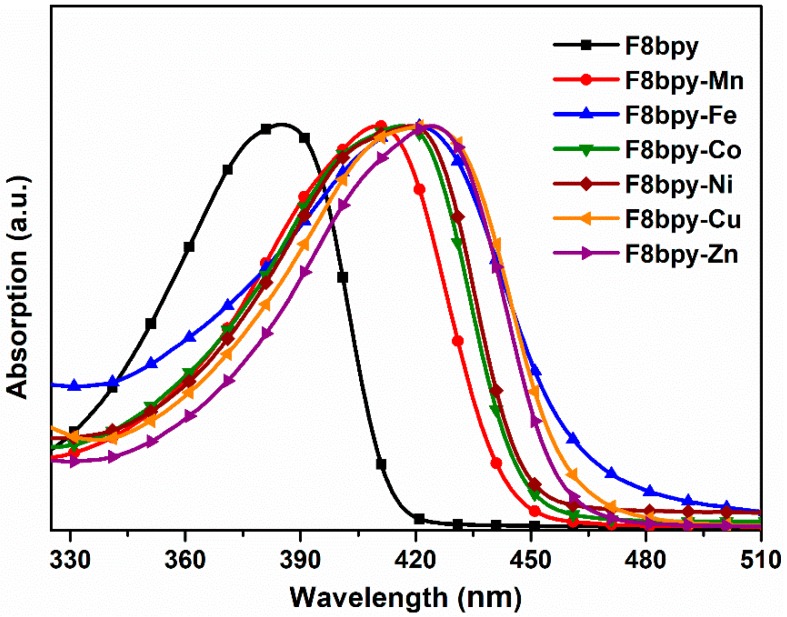
UV-vis absorption spectra of F8bpy/metal complex in chlorobenzene.

UV-vis spectra were recorded to confirm the successful complexion of the F8bpy and transition metals, as shown in Figure 3. After the formation of F8bpy-metal complexes, the absorption spectra significantly shifted to long wavelengths, indicating that the band-gaps of the F8bpy/metal complexes were much smaller than that of F8bpy itself, and the band-gap of the polymers could be tuned by coordination with different metals.

### 3.4. Characterizations of the F8bpy/Metal Complex/SWCNT Composites

**Figure 4:**
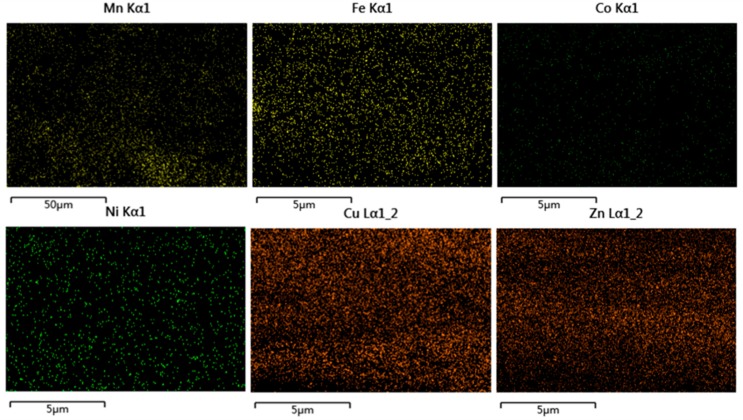
Energy dispersive X-ray spectroscopy (EDS) images of F8bpy/metal/SWCNT composites films with different transition metal ions.

The section morphologies of the F8bpy/metal complex/SWCNT composites (50/50, *w/w*) were examined by FE-SEM (Figure S3). The SWCNTs were also well dispersed in the polymer matrix. In order to further prove the uniformly dispersed state of two constituents from F8bpy/metal complex/SWCNT composites, the EDS spectra of the film cross-section were performed as shown in Figure 4. Metal ions were well dispersed in the composites, indicating that F8bpy/metal complexes were also well dispersed in the SWCNT networks.

### 3.5. Thermoelectric Performance of the F8bpy/SWCNT Composites

**Figure 5:**
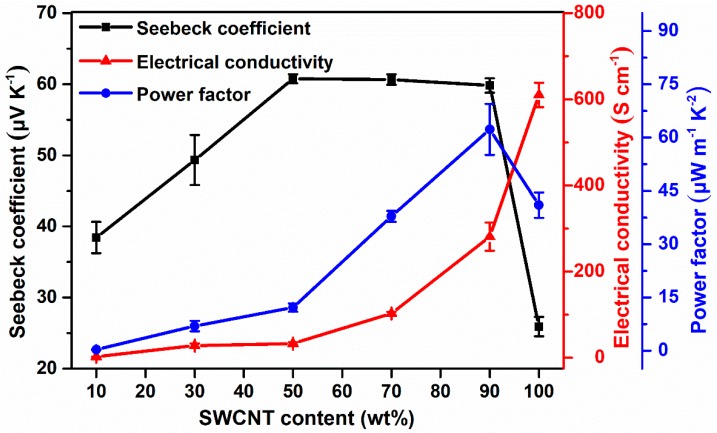
Electrical conductivities, Seebeck coefficients, and power factors at room temperature for the pure F8bpy/SWCNT composite films and pure SWCNT film.

The electrical conductivities, Seebeck coefficients, and power factors for the F8bpy/SWCNT composites films were determined by using the MRS-3 thermoelectric measurement system (Figure 5). We also attempted to measure the conductivities of pure F8bpy films by using the four-probe method, but it was too low to be determined. After the introduction of SWCNTs, the electrical conductivities of the composites were monotonously enhanced as the amount of SWCNTs was increased. Interestingly, the Seebeck coefficients of the composites also gradually enhanced with increasing the conductivities, and saturated around the mass ratio of 50/50. This phenomenon is quite rare in most of the reported thermoelectric composites, since the introduction of SWCNTs generally increased the carrier concentration of the composites, and thus the Seebeck coefficients were basically reduced, as the amount of SWCNTs increased [2]. The main reason leading to this abnormal phenomenon was probably due to the uniform wrapping of the polymer on the surface of SWCNTs with high conductivity. The homogeneous SWCNT networks of the composites also enhanced the charge carrier mobility, leading to higher conductivities and larger Seebeck coefficients. However, over a specific mass ratio of SWCNTs (for example, 50/50, *w/w*), the charge carrier concentration was still greatly enhanced, the increasing rate of the charge carrier mobility might be decreased, and the Seebeck coefficient was maintained constant while the conductivities of the composites were increased. This finding provides new strategies for simultaneously enhancing the electrical conductivities and Seebeck coefficients, which have long been considered a difficult task.

The dramatic enhancement of the electrical conductivities and Seebeck coefficients with increasing the SWCNTs mass ratios has led to the remarkable increase of the room temperature power factors with the maximum value of 62.3 μW m^−1^ K^−2^ for the F8bpy/SWCNT composites with a mass ratio of 10/90.

### 3.6. Thermoelectric Performance of the F8bpy/Metal Complex/SWCNT Composites

**Figure 6:**
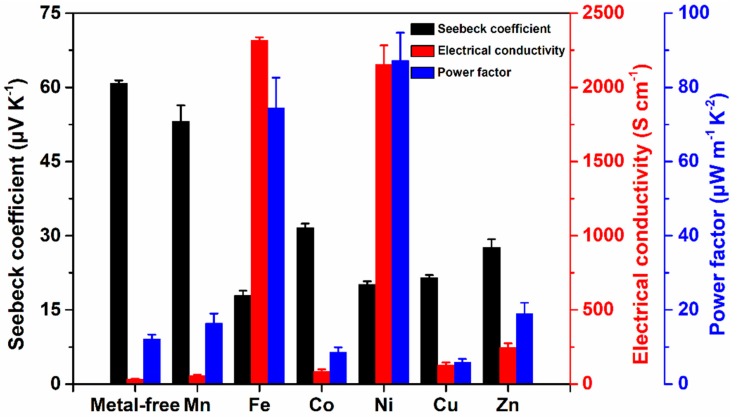
Thermoelectric properties of F8bpy/metal complex/SWCNT composite films with different transition metal ions (SWCNT 50 wt.%).

All of the F8bpy-metal complexes have low electrical conductivities, which is difficult to be determined using four-probe methods. Composites of F8bpy/metal complexes/SWCNTs with the same mass ratios (50/50) were prepared and their electrical conductivities, Seebeck coefficients, and power factors were determined by using the MRS-3 system at room temperature (Figure 6). All of the F8bpy/metal complex/SWCNT composites showed higher electrical conductivities than those of the corresponding metal-free composites. This means that the transition metals act as chemical dopants to the polymer backbone, and charger transfer to the polymer backbone occurred after chelating metals to the bipyridine ligand. The electrical conductivities of the F8bpy/Fe complex/SWCNT composites and F8bpy/Ni complex/SWCNTs were enhanced to be 2517.8 S cm^−1^ and 2153.4 S cm^−1^. This is about 70 times higher than that of a metal-free F8bpy/SWCNTs composite. The Seebeck coefficients of all the F8bpy/metal complexes/SWCNT composites decreased as the electrical conductivities increased, which should be attributed to the enhanced carrier concentration caused by the chelating of transition metals. The best power factor of 87.3 μW m^−1^ K^−2^ at room temperature was obtained for the composites of F8bpy/Ni complex/SWCNTs with a mass ratio of 50/50, and F8bpy/Fe complex/SWCNTs with a mass ratio of 50/50 exhibited a power factor of 74.5 μW m^−1^ K^−2^ at room temperature. These values are much higher than the power factor values of metal-free composites, including the maximum value of 62.3 μW m^−1^ K^−2^ for the F8bpy/SWCNT composites with a mass ratio of 10/90. We compared the thermoelectric properties of F8bpy/Ni complex/SWCNTs and other conjugated polymer/inorganic composites as shown in Table S2. The F8bpy/Ni complex/SWCNT film exhibited good thermoelectric performance among those composite systems.

## 4. Conclusions

In summary, we have developed a series of composites with SWCNTs and bipyridine-containing polyfluorene derivatives. The bipyridine ligand attached to the polymer backbone has the ability to chelate several transition metals, thus a variety of composites with different transition metals could be obtained. The thermoelectric performance of the composites could be easily tuned by changing the transition metals, and F8bpy/Ni/SWCNTs with a mass ratio of 50/50 showed a high electrical conductivity of 2153.4 S cm^−1^, which is about 70 times higher than that of metal-free F8bpy/SWCNTs with the same mass ratio, leading to the largest power factor of 87.3 μW m^−1^ K^−2^ at room temperature. This research opens a new strategy to develop transition metal complex thermoelectric materials.

## Supplementary Materials

The following are available online at http://www.mdpi.com/2073-4360/11/2/278/s1, Figure S1: ^1^H NMR spectra of polymer F8bpy, Figure S2: GPC curve of polymer F8bpy, Figure S3: Section morphology SEM images of F8bpy/metal complex/SWCNT composite films with different transition metal ions: (a) Mn, (b) Fe, (c) Co, (d) Ni, (e) Cu, (f) Zn, Figure S4: Photograph of composite film (polymer/SWCNTs with a mass ratio of 50/50) in bending state, Figure S5: (a) Photograph of MRS-3 thin film thermoelectric test system, (b) schematic diagram of electric conductivity testing for sample, (c) fitting curve of Seebeck coefficient result, (d) fitting curve of electrical conductivity result, Table S1: Thickness of the composite and SWCNT films, Table S2: The thermoelectric performance for some conjugated polymer/inorganic thermoelectric composites at room temperature.
