# Supplementary material for: Preparation and Thermoelectric Properties Study of Bipyridine-Containing Polyfluorene Derivative/SWCNT Composites
Source: Polymers (Basel). 2019 Feb 7;11(2):278. doi: 10.3390/polym11020278 (PMC6419042; doi:10.3390/polym11020278)
Supplement: Supplementary file 1 [file polymers-11-00278-s001.pdf]

polymers

MDPI

Supporting Information

Preparation and Thermoelectric Properties Study of Bipyridine-Containing Polyfluorene Derivative/SWCNT Composites

Chengjun Pan*[, 1], Luhai Wang[1], Wenqiao Zhou[1], Dexun Xie[3], Lirong Cai[2], Zhongming Chen*[, 2] and Lei Wang*[, 1]

[1] Shenzhen Key Laboratory of Polymer Science and Technology, College of Materials Science and Engineering, Shenzhen University, Shenzhen 518060, China
[2] School of Environment and Civil Engineering, Dongguan University of Technology, Dongguan 523808, China.
[3] School of Chemistry, Sun Yat-sen University, Guangzhou, 510275, China; Shenzhen Research Institute, Sun Yat-sen University, Shenzhen, 518057, China.
* Email: pancj@su.edu.cn; zmchen@dgut.edu.cn； wl@szu.edu.cn

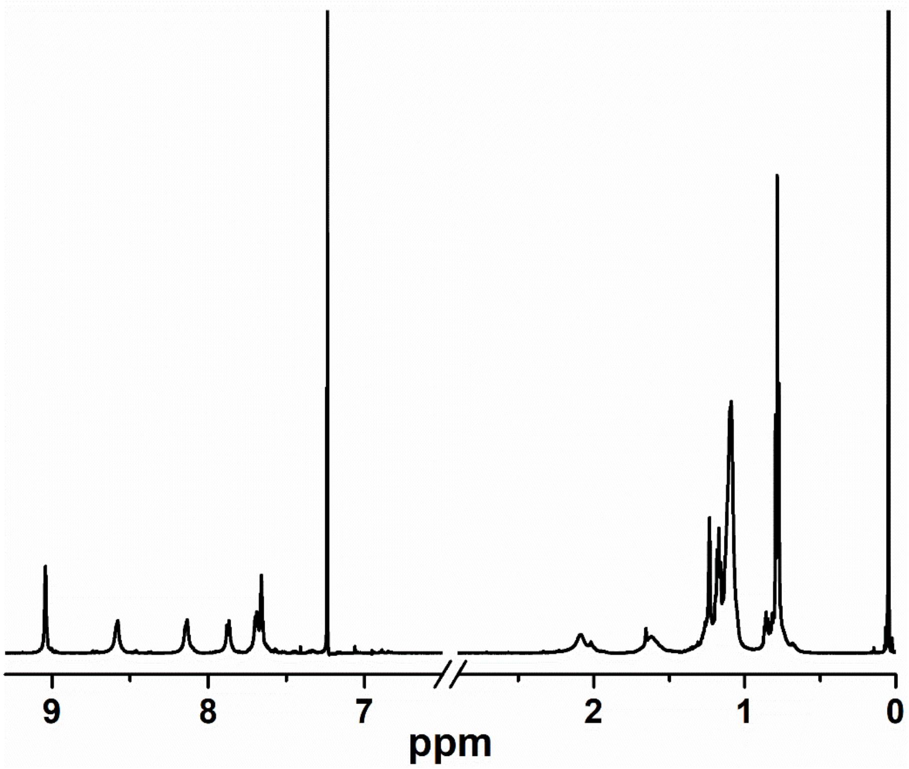

Figure S1. ^{1}H NMR spectra of polymer F8bpy.

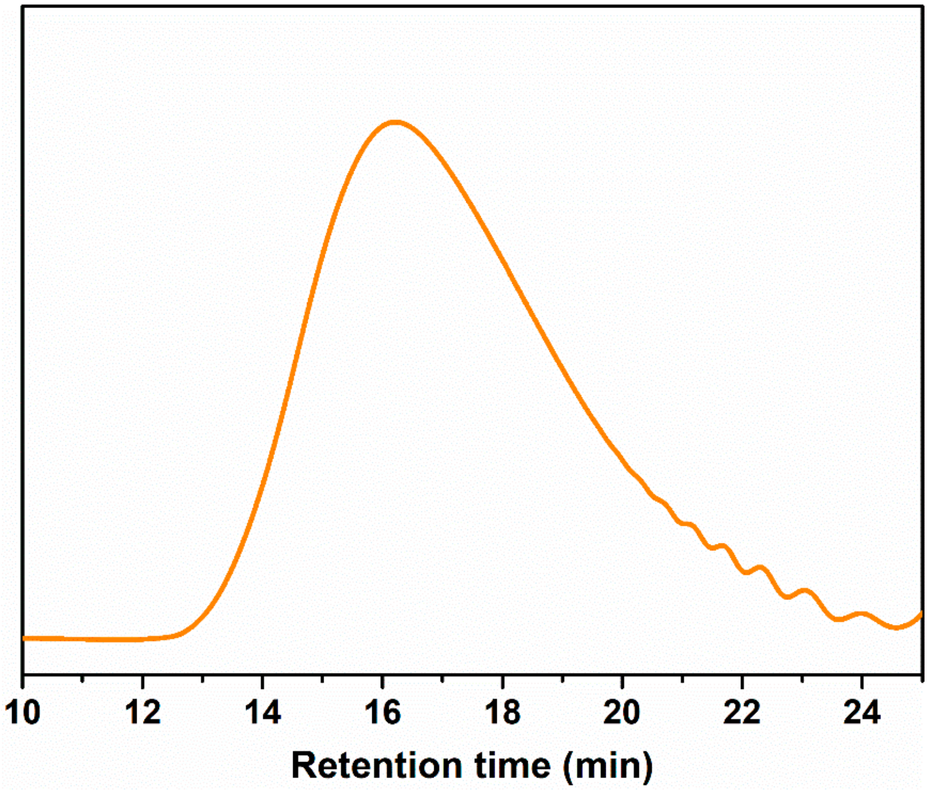

Figure S2. GPC curve of polymer F8bpy.

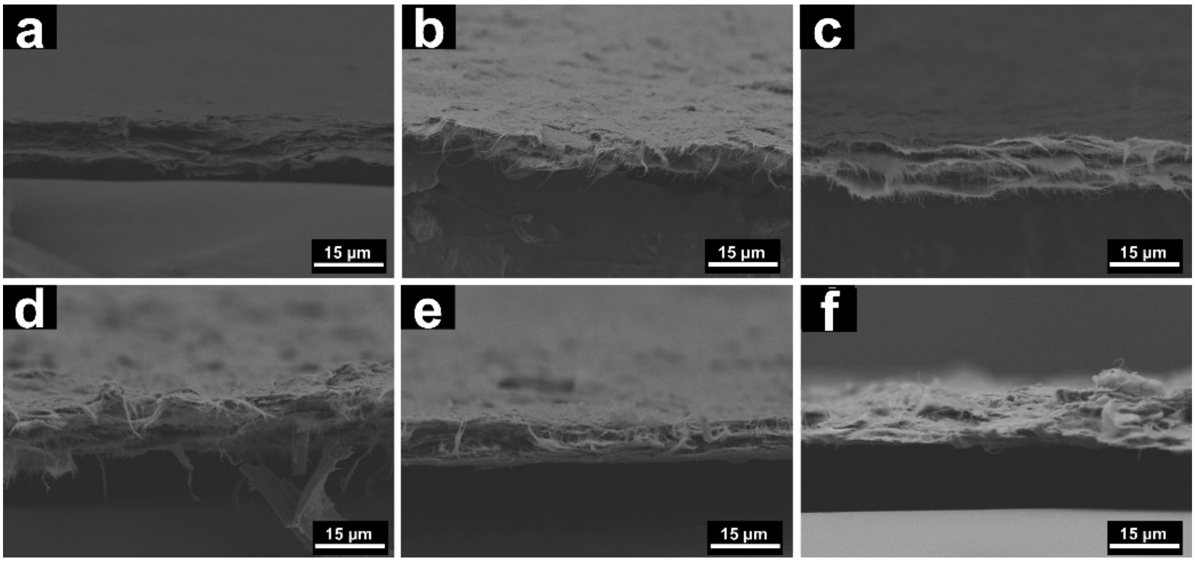

Figure S3. Section morphology SEM images of F8bpy/metal complex/SWCNT composite films with different transition metal ions: (**a**) Mn, (**b**) Fe, (**c**) Co, (**d**) Ni, (**e**) Cu, (**f**) Zn.

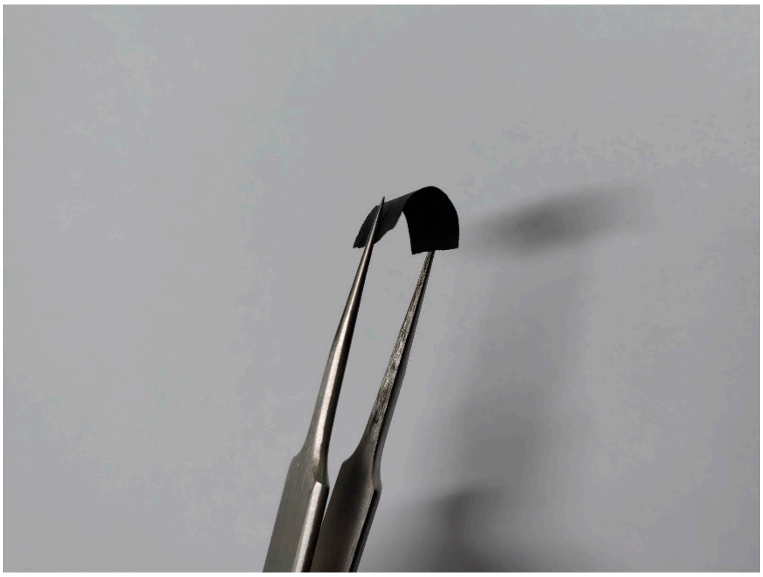

Figure S4. Photograph of composite film (polymer/SWCNTs with a mass ratio of 50/50) in bending state.

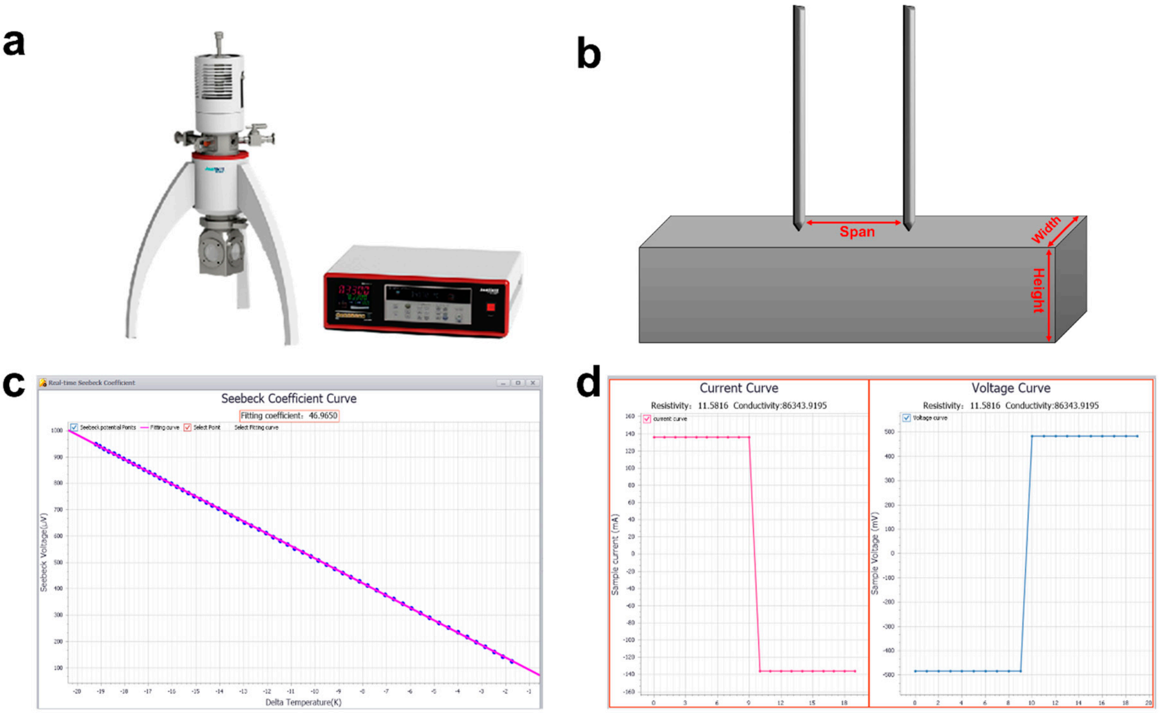

Figure 5. (**a**) Photograph of MRS-3 thin film thermoelectric test system. (**b**) Schematic diagram of electric conductivity testing for sample. (**c**) Fitting curve of Seebeck coefficient result. (**d**) Fitting curve of electrical conductivity result.

Figure S5a shows the photograph of MRS-3 thin film thermoelectric test system; the left is the device for placing samples, and the right is the test host. Figure S5b exhibits the sample figure for electrical conductivity testing. Figures S5c,d show the fitting curve of the Seebeck coefficient and the electrical conductivity results. Based on the basic principle of testing the material's Seebeck coefficient, this device adopts a patented quasi-dynamic method to measure the Seebeck coefficient. The principle of the measurement of Seebeck coefficient is as follows. In the program by heating one

end of sample for a period of time, a series of gradually increasing temperature differences are formed between the cold and hot ends of the sample ($\triangle T_1$, $\triangle T_2$ … $\triangle T_n$). Then, the corresponding temperature difference potentials ($\triangle V_1$, $\triangle V_2$ … $\triangle V_n$) are collected. Based on these obtained data, and according to the least square method, ininzum residual method, or BiSquare method to obtain the slope *K* of measured sample, the slope *K* is the Seebeck coefficient. The principle of measurement of electrical conductivity is as follows: Based on the formula $\rho = V \times A/I \times L$ and $\sigma = 1/\rho$, where ρ and σ are the resistivity and electrical conductivity of the sample, *V* and *I* are respectively the current and the detection terminal voltage in the detection circuit; *A* and *L* are the energized cross-sectional area (Width × Height) and potential detection distance (Span).

Table 1. Thickness of the composite and SWCNT films.

Samples	Thickness (μm)
F8bpy/SWCNTs (10/90)	6.98 ± 0.76
F8bpy/SWCNTs (30/70)	10.05 ± 1.32
F8bpy/SWCNTs (50/50)	8.70 ± 0.56
F8bpy/SWCNTs (70/30)	12.50 ± 1.23
F8bpy/SWCNTs (90/10)	10.25 ± 0.96
SWCNTs	11.62 ± 0.83
F8bpy-Mn/SWCNTs	7.90 ± 0.65
F8bpy-Fe/SWCNTs	6.21 ± 0.66
F8bpy-Co/SWCNTs	7.83 ± 0.78
F8bpy-Ni/SWCNTs	8.71 ± 0.46
F8bpy-Cu/SWCNTs	7.05 ± 0.93
F8bpy-Zn/SWCNTs	5.42 ± 0.39

Table 2. The thermoelectric performance of some conjugated polymer/inorganic thermoelectric composites at room temperature.

Polymer	Inorganic	S (μV K^{-1})	E (S cm^{-1})	PF (μW m^{-1} K^{-2})	Ref.
PANI	Graphene	26	814	55	1
PEDOT:PSS	SWCNT	44.3	535	105	2
PPy	SWCNT	22.2	399	19.7	3
P3HT	Graphene	35.46	1.27	0.16	4
PEDOT:PSS	Te/SWCNT	56	332	104	5
PANI	Bi2Te3	102.22	28.06	29.32	6
PPy/PANI	Graphene	500	32	52.5	7
PPy	Reduced graphene	29	86.5	7.28	8
F8bpy-Ni	SWCNT	20.1	2153.4	87.3	This work

References

1. Wang, L.; ao, Q.; Bi, H.; Huang, F.; Wang, Q.; Chen, L. PANI/graphene nanocomposite films with high thermoelectric properties by enhanced molecular ordering. *J. Mater. Chem. A* **2015**, 3, 7086-7092.

2. Jiang, Q.; Lan, X.; Liu, C.; Shi, H.; Zhu, Z.; Zhao, F.; Xu, J.; Jiang, F. High-performance hybrid organic thermoelectric SWNTs/PEDOT: PSS thin-films for energy harvesting. *Mater. Chem. Front.* **2018**, 2, 679-685.

3. Liang, L.; Gao, C.; Chen, G.; Guo, C.-Y. Large-area, stretchable, super flexible and mechanically stable thermoelectric films of polymer/carbon nanotube composites. *J. Mater. Chem. C* **2016**, 4, 526-532.

4. Du, Y.; Cai, K. F.; Shen, S. Z.; Casey, P. S. Preparation and characterization of graphene nanosheets/poly(3-hexylthiophene) thermoelectric composite materials. *Synth. Met.* **2012**, 162, 2102-2106.

5. Meng, Q. F.; Cai, K. F.; Du, Y.; Chen, L. D. Preparation and thermoelectric properties of SWCNT/PEDOT:PSS coated tellurium nanorod composite films. *J. Alloys Compd.* **2019**, 778, 163-169.
6. Mitra, M.; Kulsi, C.; Kargupta, K.; Ganguly, S.; Banerjee, D. Composite of polyaniline-bismuth selenide with enhanced thermoelectric performance. *J. Appl. Polym. Sci.* **2018**, 135, 46887.
7. Wang, Y. H.; Yang, J.; Wang, L. Y.; Du, K.; Yin, Q.; Yin, Q. J. Polypyrrole/Graphene/Polyaniline Ternary Nanocomposite with High Thermoelectric Power Factor. *ACS Appl. Mater. Interfaces* **2017**, 9, 20124-20131.
8. Xin, S.; Yang, N.; Gao, F.; Zhao, J.; Li, L.; Teng, C. Free-standing and flexible polypyrrole nanotube/reduced graphene oxide hybrid film with promising thermoelectric performance. *Mater. Chem. Phys.* **2018**, 212, 440-445.

© 2018 by the authors. Submitted for possible open access publication under the terms and conditions of the Creative Commons Attribution (CC BY) license (http://creativecommons.org/licenses/by/4.0/).
